# Evaluating Amyloid-β Oligomers in Cerebrospinal Fluid as a Biomarker for Alzheimer’s Disease

**DOI:** 10.1371/journal.pone.0066381

**Published:** 2013-06-14

**Authors:** Mikko Hölttä, Oskar Hansson, Ulf Andreasson, Joakim Hertze, Lennart Minthon, Katarina Nägga, Niels Andreasen, Henrik Zetterberg, Kaj Blennow

**Affiliations:** 1 Institute of Neuroscience and Physiology, Department of Psychiatry and Neurochemistry, The Sahlgrenska Academy at University of Gothenburg, Mölndal, Sweden; 2 Clinical Memory Research Unit, Department of Clinical Sciences Malmö, Lund University, Malmö, Sweden; 3 Department of Clinical Neurosciences and Family Medicine, Section of Geriatric Medicine, Karolinska University Hospital, Stockholm, Sweden; 4 UCL Institute of Neurology, University College London, London, United Kingdom; Federal University of Rio de Janeiro, Brazil

## Abstract

The current study evaluated amyloid-β oligomers (Aβo) in cerebrospinal fluid as a clinical biomarker for Alzheimer’s disease (AD). We developed a highly sensitive Aβo ELISA using the same N-terminal monoclonal antibody (82E1) for capture and detection. CSF samples from patients with AD, mild cognitive impairment (MCI), and healthy controls were examined. The assay was specific for oligomerized Aβ with a lower limit of quantification of 200 fg/ml, and the assay signal showed a tight correlation with synthetic Aβo levels. Three clinical materials of well characterized AD patients (n = 199) and cognitively healthy controls (n = 148) from different clinical centers were included, together with a clinical material of patients with MCI (n = 165). Aβo levels were elevated in the all three AD-control comparisons although with a large overlap and a separation from controls that was far from complete. Patients with MCI who later converted to AD had increased Aβo levels on a group level but several samples had undetectable levels. These results indicate that presence of high or measurable Aβo levels in CSF is clearly associated with AD, but the overlap is too large for the test to have any diagnostic potential on its own.

## Introduction

Alzheimer’s disease (AD) is the most common form of dementia affecting more than 15 million people in the world and is characterized by progressive neuronal degeneration with depositions of amyloid plaques and neurofibrillary tangles [1]. The amyloid plaques have been shown to mainly consist of aggregated amyloid-β (Aβ) 1–42, while the neurofibrillary tangles consist of aggregated phosphorylated tau [2], [3]. The pathological process is believed to begin 10–20 years before the first clinical symptoms arise, with amyloid plaque formation starting in the neocortex and can later on be seen throughout the brain [4]. As an intermediate state before Aβ forms plaques, small soluble aggregates called Aβ oligomers (Aβo) are believed to be formed [5], [6], [7]. Animal studies in rodents have shown that small soluble Aβo impair memory [8], affect long term potentiation [9], and lead to cognitive deficits [10]. The neurotoxic effects of Aβo appear to involve modulation of the NMDA receptor and metabotropic glutamate receptors and possibly also pore formation in membranes [11], [12], [13], [14]. The neurotoxic effect can be reversed in rodents by using immunotherapy against Aβ and by inhibiting Aβ oligomerization with peptides [15], [16], [17], [18].

Today, three established cerebrospinal fluid (CSF) biomarkers are used to aid the diagnosis of AD; increased phosphorylated tau (P-tau_181_), increased total tau (T-tau), and decreased Aβ_1–42_, for review see [19]. Several studies have demonstrated that Aβ_1–42_ levels are decreased in AD patients compared to healthy controls, and this is also reported in patients with prodromal AD [20], [21], [22]. Amyloid plaques in the brain can be visualized by positron emission tomography (PET), using the ligand ^11^C-PIB, which binds to fibrillar Aβ [23]. The belief is that the lowering of Aβ_1–42_ is caused by its incorporation into plaques, which is consistent with studies showing that high ^11^C-PIB binding correlates with lower levels of Aβ_1–42_ in CSF [24], [25]. If this lowering is caused by Aβ oligomerization and aggregation, Aβo would potentially be an early biomarker for AD reflecting an ongoing pathology.

In CSF, Aβo has been measured with various techniques [26], [27], [28], [29]. Fukumoto and co-workers recently showed high CSF levels of Aβo in AD patients using and assay based on the monoclonal antibody BAN50 both for capture and detection and synthetic Aβo as standard [30]. Using flow cytometry, Santos and co-workers [31] showed that there was a trend of elevated Aβo levels in AD patients compared to controls and Gao and co-workers [32] also found increased levels of oligomeric Aβ_1–40_ in CSF using a novel misfolded protein assay. Using nanoparticle detection an increase in amyloid-β-derived diffusible ligands has also been reported [29].

In this study, we developed a sandwich ELISA using the same N-terminally specific Aβ antibody as both capture and detection antibody to measure Aβo in CSF. N-terminally specific antibodies have been demonstrated to have higher affinity against fibrillar Aβ than antibodies with an epitope against the more C-terminal part of the Aβ sequence [33], [34], indicating that the N-terminal part of the Aβ sequence is the most likely one to be exposed in Aβ aggregates. We compared four patient materials with AD patients to healthy controls, and also a longitudinal mild cognitive impairment (MCI) cohort, to evaluate whether Aβo measured with this type of assay could be used as a clinical biomarker.

## Materials and Methods

### Participants

Four study populations were recruited at three specialized and coordinated memory clinics in Sweden within the Swedish Brain Power network (Malmö, Stockholm and Piteå). The Piteå and Stockholm centers are run by the same clinician with identical sampling and storage protocols and are hence considered as one center. Demographics and biochemical characteristics are given in Table 1. A set of nine younger controls from the Malmö clinic, median age 42, were included to study possible age effects. All subjects underwent an extensive clinical examination, also including cognitive evaluations with mini-mental state examination (MMSE) [35]. All patients also underwent imaging of the brain and lumbar puncture for CSF collection.

**Table 1:** Demographics and biomarker concentrations for all AD patients, MCI patients and controls.

Study	Diagnosis	Nr	Sex (M/F)	Age (years)	Aβ_1–42_ (pg/ml)	P-tau_181_(pg/ml)	T-tau (pg/ml)	MMSE	Aβo (fg/ml)
I	Control	31	16/15	61 (52, 67)	690 (466, 898)	47 (33, 61)	285 (188, 365)	29 (28, 29)	522 (339, 781)
	AD	42	10/32	79 (74, 81)***	370 (318, 415)***	92 (80, 116)***	770 (640, 895)***	21 (19, 23)***	1040 (773, 1,303)***
II	Control	22	10/12	69 (66, 72)	780 (645, 1,060)	–	405 (178, 500)	30 (30, 30)	0 (0, 0)
	AD	51	22/29	79 (75, 81)***	440 (326, 508)***	–	623 (465, 858)***	23 (20, 26)***	717 (0, 1,490)***
III	Control	62	17/45	74(68, 78)	298 (237, 342)	33 (23, 41)	79 (56, 97)	29 (28, 30)	0 (0, 0)
	MCI-AD	58	21/37	78(73, 81)	146 (119, 177)***	49 (36, 67)***	129 (93, 181)**	26 (25, 27)***	0 (0, 313)**
	MCI-Stable	77	34/43	67(62, 75)***	266(217, 298)**	27(19, 36)*	71 (50, 90)	28 (28, 29)**	0 (0, 295)
IV	Control	33	13/20	69 (62, 72)	867 (737, 1060)	–	383 (250, 502)	30 (30, 30)	1538 (504, 2408)
	Severe	11	7/4	79 (68, 82)**	398 (366, 400)***	–	937 (648, 1270)***	12 (13, 16)***	1250 (576, 2082)
	Moderate	51	21/30	80 (75, 83)***	435 (370, 494)***	–	760 (595, 886)***	21 (20, 23)***	2647 (1395, 3451)***
	Mild	44	18/26	77 (74, 81)***	473 (440, 524)***	–	848 (631, 899)***	27 (26, 28)***	2467 (1250, 3387)**

Data are given as medians with 25th and 75th percentiles.

* *p*<0.05,

** *p*<0.01,

*** *p*<0.001 vs. control group.

The analyses of Aβ_1–42_, P-tau_181_ and T-tau have been performed with ELISA earlier [22], [48], [49], [50].

Controls had no history or clinical signs of neurological or psychiatric disease or cognitive symptoms. AD was diagnosed following the criteria for probable AD according to the National Institute of Neurological and Communicative Disorders and Stroke- Alzheimer’s Disease and Related Disorders Association (NINCDS-ADRDA) [36]. Disease severity was evaluated using MMSE scores and mild patients had a MMSE of 25–30; moderate AD patients had a MMSE of 17–24, and severe AD patients had a MMSE of 16 or lower. MCI was diagnosed in patients with cognitive impairment that did not fulfill the criteria for dementia [37]. During clinical follow-up of the patients with MCI at baseline, 35% developed AD and 30% developed other forms dementia disorders, but 47% were cognitively stable for a median time of 6.3 years (range 3.0y to 9.6y).

CSF collection was conducted following standardized operating procedures [19]. Lumbar puncture was performed in the L3–L4 or L4–L5 interspace. The first 12 mL of CSF was collected in a polypropylene tube and was centrifuged at 2000×g at 4°C for 10 min. The supernatant was pipetted off, gently mixed to avoid possible gradient effects, and aliquoted in polypropylene tubes that were stored at −80°C pending biochemical analyses.

### Ethics Statement

The studies were approved by the ethics committees at Lund University, Umeå University and Karolinska Institute. The participants provided their verbal informed consent for research, documented in the patient journals, which is the standard procedure in Sweden and approved by the ethics committees.

### Amyloid-β Oligomer ELISA

**Figure 1:**
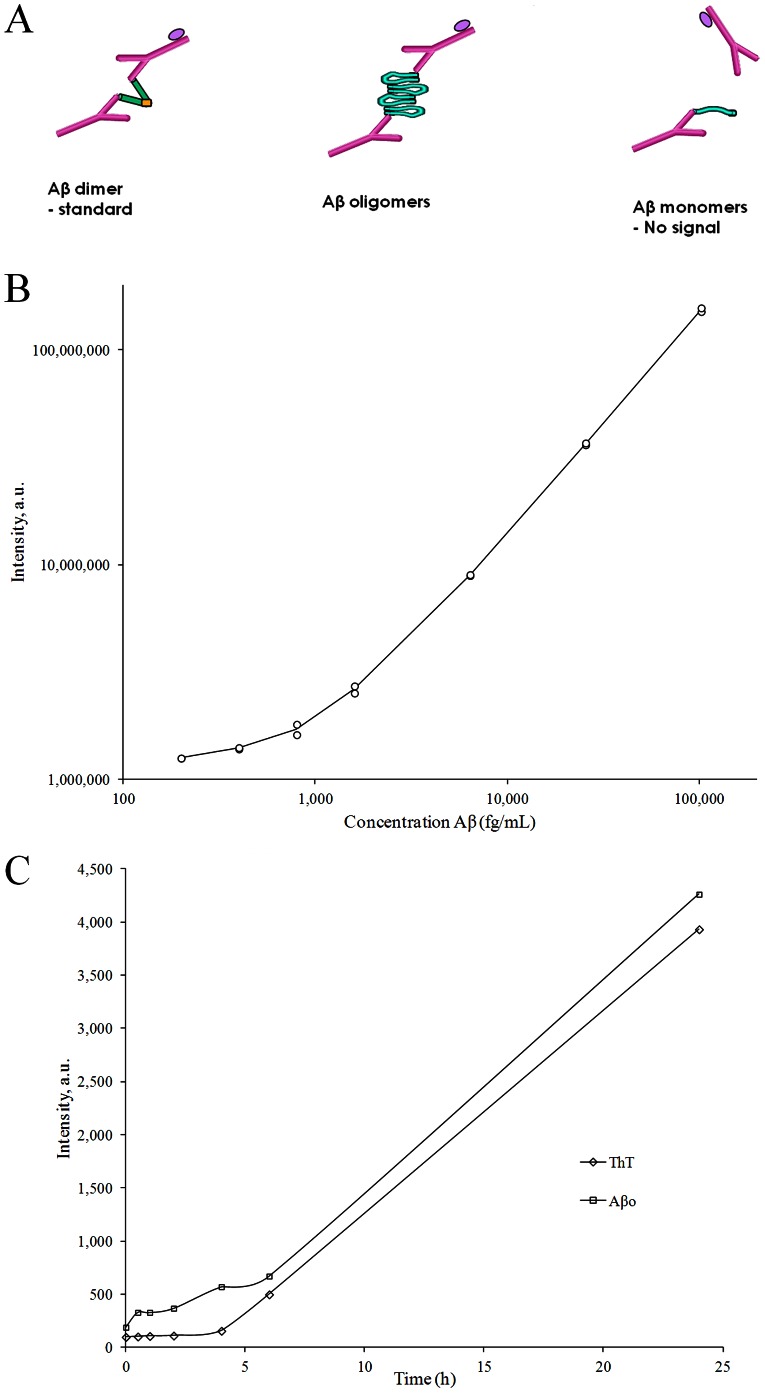
ELISA method for Aβ oligomers in cerebrospinal fluid. A) Schematic drawing of the principle for the method. Left: The Aβo ELISA is based on the use of the same N-terminal anti-Aβ monoclonal antibody twice. The ELISA plate is coated with 82E1 to capture all forms of Aβ, while biotinylated 82E1 is used for detection. A synthetic Aβ dimer, with two N-termini, is used as standard. Middle: Aβos, with several free N-terminals, are detected in the assay. Right: monomeric Aβ will have their epitopes blocked by the capture antibody and are thus not detected by the detection antibody. B) Example of a typical standard curve from the Aβo assay. The standard curve ranges from 200–102,400 fg/mL. The assay has a lower limit of quantification of 200 fg/mL. C) Measurement of synthetic Aβo formation by the Aβo ELISA. Synthetic Aβ_1–42_ was allowed to aggregate into Aβ oligomers. The signal in the Aβo ELISA was compared with a Thioflavin-T (ThT) assay for aggregated Aβ. The Aβo ELISA detects the formation of synthetic Aβo at an earlier stage than the ThT assay, while following the increase of oligomerization in parallel with the ThT assay after 5 hours.

For the Aβo ELISA the Aβ N-terminal specific antibody 82E1 [38] (IBL international, Hamburg, Germany) was used both for capture and detection. The use of the same monoclonal antibody for capture and detection has been demonstrated in previous studies to specifically detect aggregated forms of Aβ without detecting monomers [30], [39], [40], [41]. A synthetic dimer consisting of two Aβ_1–11_ peptides with an added C-terminal cysteine through which the peptides were coupled via a disulfide bridge (Caslo, Denmark) was used to create the standard curve. A schematic outline of the Aβo ELISA is presented in Figure 1A.

An ELISA plate (Black MaxiSorp FluoroNunc, Nunc, Denmark) was coated with 82E1 diluted in 50 mM NaHCO_3_, pH 9.6 to a concentration of 1 µg/ml, 100 µl/well, over night in +4°C. The plate was washed 5 times with 350 µl phosphate buffered saline containing 0.05% Tween20 (Bio-Rad) (PBST). Blocking was done using 2% bovine serum albumin (BSA) (Sigma Aldrich) dissolved in PBST, 300 µl/well at room temperature (RT) for 1 h. The plate was then washed 5 times with PBST. The standard was prepared by dilution of the synthetic dimer in 0.1% BSA-PBST, 200–102,000 fg/ml. CSF samples and standards were added in duplicates, 100 µl/well and incubated for 1 h at RT, where after the plate was washed 5 times with PBST. Detection antibody, biotinylated 82E1, diluted in 0.1% BSA-PBST to a concentration of 750 ng/ml, was added, 100 µl/well, and incubated for 1 h in RT. The plate was washed 5 times with PBST. NeutrAvidin horseradish peroxidase conjugate (Thermo Scientific), diluted 1∶5,000 in 0.1% BSA-PBST, was added, 100 µl/well, and incubated for 1 h at RT, and the plate was washed 5 times with PBST. For detection SuperSignal Femto maximum sensitivity substrate (Thermo Scientific, Pierce Biotechnology, Rockford, Illinois, USA) 100 µl/well was used, read 1 s/well on a Victor X4 (Perkin Elmer). The read-out data from Victor X4 were analyzed with SoftMax Pro 4.7.1 (Molecular Devices).

The detection was done using a colorimetric method for the oligomerization study and on the analyses of brain tissue. TMB substrate (Bio-Rad), 100 µl/well was incubated at RT for 15 min, and the reaction was stopped with 100 µl of 2 M H_2_SO_4_. Absorbance was measured at 450 nm using a V-Max microplate reader (Molecular Devices).

All samples for each individual study were analyzed on the same day.

### ThT Oligomerization Assay

To initiate the oligomerization, a buffer solution containing ThT (Sigma-Aldrich) was added to synthetic Aβ_1–42_ (Anaspec), reconstituted in 10 mM NaOH. The final concentrations were 200 mM HEPES, pH 8, 40 µM ThT, and 40 µM Aβ_1–42_. The mixture was transferred to a black 384-well microtiter plate (Nunc, Denmark) and overlaid with mineral oil (Sigma-Aldrich) to prevent evaporation. Fluorescence was measured at 37°C in kinetic mode every 30 min using a SpectraMax Gemini XPS (Molecular Devices) with excitation and emission wavelengths of 450 nm and 485 nm, respectively. One vial with the exact same reagents was in parallel kept in 37°C from which samples were taken at given time points, quickly frozen, and stored at −80°C pending the Aβo ELISA analysis.

### Cross Reactivity

To test the cross reactivity to monomeric Aβ in the Aβo ELISA, Aβ_1–16_ was diluted in 0.1% BSA-PBST in concentrations up to 100,000 pg/ml, while Aβ_1–40_ was diluted up to 5,000 pg/ml and measured with the Aβo ELISA.

### Brain Tissue from AD Patients and Tg2576 Mice

Cortical tissue from Tg2576 mice were prepared as described previously [42]. In brief the brain cortices were homogenized in a Tris buffer and centrifuged at 16.000×g where after the supernatants were collected and analyzed. Human AD brain was homogenized in Tris buffer containing Complete Proteasinhibitor (Roche, Protease Inhibitor Cocktail tablets) and 0.5% Triton x-100, and centrifuged at 30,000×g for 60 min at 4°C. The supernatant was analyzed.

### Freeze/thawing Experiments

CSF samples (n = 4) were thawed and kept in room temperature and then re-frozen at −80°C in 5 cycles. An identical sample was kept in −80°C that did not undergo the freeze/thaw cycles to be used as control.

### Heterophilic Antibodies

A set of eight CSF samples, with Aβo concentration range of 600–4800 fg/mL, were used to evaluate whether heterophilic antibodies interfere with the assay. The CSF samples were mixed with mouse IgG (Sigma-Aldrich, I5381) to a concentration of 10 µg/mL and incubated for 30 minutes before being analyzed together with the same samples without added mouse IgG.

### Molecular Weight Filtration of Oligomers

Synthetic Aβo generated according to Berghorn et al [43] were sequentially spun through different sized molecular weight cut-off filters (Amicon ultra, Millipore), 50 kDa, 30 kDa, and 10 kDa and the fractions from these were analyzed.

### Statistics

Statistical analyses were performed with SPSS PASW 18 (SPSS Inc, Chicago, Illinois, USA), using nonparametric tests because of skewed distribution in the variables. For comparisons between groups Mann Whitney U-test was used, and data are presented as median with interquartile range. Correlation analyses were done using the Spearman correlation coefficient. Scatter plots were done using GraphPad Prism v5.02 (GraphPad Software Inc, La Jolla, California, USA).

## Results

### Aβo ELISA Characteristics

The standard curve used in the Aβo assay ranged from 200 fg/mL to 102,400 fg/mL, Figure 1B. The limit of quantification was determined to 200 fg/mL by calculating the Aβo concentration at 10 standard deviations above the blank.

The specificity for the assay was tested using oligomerized Aβ_1–42_, generated according to the protocol developed by Berghorn et al [43], which followed a titration curve. In contrast, spiking with monomeric Aβ_1–16_ up to 100,000 pg/ml or monomeric Aβ_1–40_ up to 5,000 pg/ml did not result in any detectable concentration. The specificity for oligomerized Aβ was also seen when a ThT assay was performed in parallel to the Aβo ELISA. As can be seen in Figure 1C there is no reaction at time point zero, with a dramatic increase between 2–24 hours. The Aβo ELISA also showed an earlier detection of Aβo formation than the ThT assay (Figure 1C). The assay was also tested for interference from heterophilic antibodies by mixing CSF with mouse IgG, allowing potential heterophilic antibodies to react with the mouse IgG and thus block their ability to cross-react with the capture and detection antibody. This showed no significant decrease in the Aβo signal.

With the Aβo assay, it was possible to measure Aβo in brain extracts from transgenic mice. The concentrations of soluble Aβo in brain cortex tissue from Tg2576 mice extracted with TBS were 106 pg Aβo/mg protein at the age of 7 days and decreased to 67 pg Aβo/mg protein at the age of 90 days. In human AD brain an Aβo concentration of 826 pg/mg protein was measured. The assay reacts with synthetic oligomers with a molecular weight above 10 kDa, showing reactivity for Aβo with a molecular weight of 10–30 kDa, 30–50 kDa, and >50 kDa.

The measured values of the freeze/thawed samples did not significantly diverge from samples that did not undergo these cycles. The coefficient of variation (CV) of the freeze/thawed sample compared to the control samples where in the range 1–18%. The intra-assay CV was less than 7% determined by measuring 7 CSF samples in duplicate. The inter-assay CV was less than 20%.

### Clinical Studies on the Diagnostic Performance of CSF Aβ Oligomers

**Figure 2:**
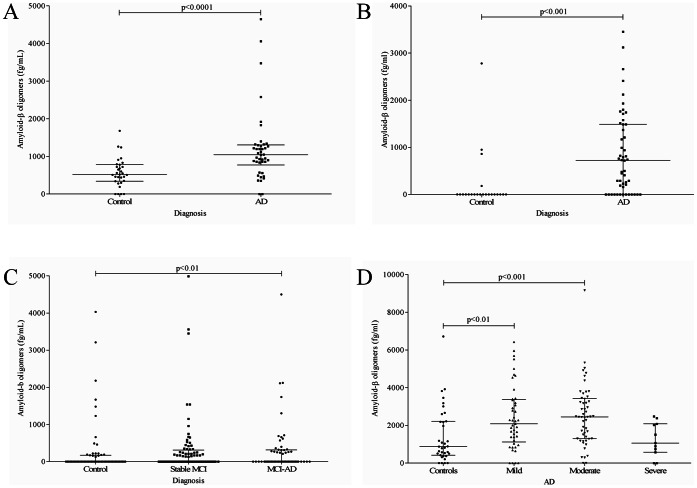
Cerebrospinal fluid Aβ oligomers in independent clinical samples. A) First AD study (Malmö). Increased CSF levels of Aβo in the AD group (n = 42) compared to the control group (n = 31), p<0.0001. Bars indicate median with interquartile range. B) Second AD study (Piteå and Stockholm). Increased CSF levels of Aβo in the group of patients with AD (n = 51) compared to the control group (n = 22), p<0.001. Bars indicate median with interquartile range. C) MCI study. Increased CSF levels of Aβo in the group of MCI patients who converted to AD during the follow-up period (n = 58) as compared to the control group (n = 62), p<0.01. No significant difference in CSF Aβo between stable MCI (p = 0.059) and controls. Bars indicate median with interquartile range. D) Clinical study on AD with different severity of dementia. Increased CSF levels of Aβo in the group of AD patients with mild (n = 44, p<0.01) and moderate (n = 51, p<0.001) dementia as compared to the control group (n = 33). No significant change was found in the AD group with severe dementia (n = 11) compared to the control group. Bars indicate median with interquartile range.

In the first clinical study, we found a significant (p<0.0001) increase in AD CSF Aβo compared to the control group (Figure 2a), 1040 fg/mL and 522 fg/mL respectively. However, there was a marked overlap between the two groups, 64% of AD patients had a CSF level of Aβo higher than the optimal cut-off of 835 fg/mL (84% specificity).

For this reason, we analyzed a second clinical study of AD patients with dementia from another clinical center. We could verify a significant (p<0.001) increase in the CSF levels of Aβo compared to the control group (Figure 2b), 717 fg/mL and <200 fg/mL respectively. However, again there was a marked overlap between the two groups, 67% of AD patients had CSF levels of Aβo higher than the optimal cut-off of 215 fg/mL (86% specificity).

We then hypothesized that there may be a more marked release of Aβo into CSF during the earlier stages of the disease. We therefore analyzed an independent clinical study including patients with MCI. In this clinical study (Figure 2C), we found that MCI patients who later converted to AD (MCI-AD) had increased levels of Aβo compared to controls, p<0.01, <200 fg/mL and 210 fg/mL respectively, while patients with stable MCI did not differ from controls, <200 fg/ml and <200 fg/mL respectively. However, there was a marked overlap between MCI-AD and controls, with only 44% of MCI-AD patients having CSF Aβo levels above the cut-off of 230 fg/mL (85% specificity).

Last, we tested the reverse hypothesis, that there may be a more marked release of Aβo into CSF during the very last stages of the disease. The basis for this hypothesis was that plaques may act as a reservoir for aggregated Aβ, which in the later stages of the disease might have reached their maximal capacity, causing Aβo to leak out from the brain into the CSF. We therefore analyzed an independent clinical study with AD patients with different severity of dementia (mild – moderate – severe) based on their MMSE scores. In this clinical study (Figure 2D) we found that AD patients with mild (2467 fg/mL) and moderate (2647 fg/mL) dementia had significantly higher levels of Aβo compared to healthy controls (1538 fg/mL), p<0.01 and p<0.001 respectively, while AD patients with severe dementia (1250 fg/mL) did not significantly differ from the control group (Figure 2d).

### Aβo in Relation to Established Biomarkers for AD

In all four studies, the levels of the three established biomarkers were significantly changed in the AD vs. control population, with decreased Aβ_1–42_, increased P-tau_181_, and increased T-tau in the AD group (Table 1). This was also seen in the MCI patients who later converted to AD (Table 1).

No significant correlations were found between Aβo and the biomarkers in any of the control, AD or MCI groups.

### Influence of Age on Aβo

The age of the subjects did not correlate with Aβo levels in any of the groups. A comparison between younger healthy controls against the healthy controls in study I, both sampled at the same clinic, did not show any significant age difference between the Aβo levels, 1160 fg/mL vs. 580 fg/mL respectively, p = 0.277.

## Discussion

In this study we evaluated if CSF Aβo could be used as a clinical biomarker for AD by analyzing four different patient materials from different clinical centers. Three patient materials were analyzed, where AD patients consistently had significantly increased levels of Aβo compared to controls. In one study stable MCI and MCI-AD patients were analyzed, which showed an increase in Aβo in MCI-AD patients but not in stable MCI patients.

We developed a highly sensitive and specific CSF Aβo ELISA, similar to the one used by Xia et al [44], where we used a synthetic Aβ dimer with two free N-terminals, instead of a preparation of aggregated Aβ_1–42_ used in earlier studies [30], [32] to create the standard curve and a chemiluminescent substrate for detection. The use of a synthetic dimer enables quantification and comparisons of results longitudinally since the dimer is stable and at known concentrations. This gives an Aβo concentration which is relative to the dimer, and the signal from a synthetic oligomer mixture correlates with the dimer concentration when titrated in parallel, why differences in Aβo levels between AD and controls won’t be affected by the use of a dimer instead of a mixture of synthetic oligomers. The Aβo ELISA was run in parallel with a ThT assay showing that the oligomerization of Aβ_1–42_ measured by the Aβo ELISA followed the results from the ThT assay. The assay detects Aβo larger than 10 kDa which includes slightly smaller Aβo than Fukumoto et al [30] detect with their assay, but the clinical relevance of these are unknown. No correlation was found between age and Aβo levels in the patient groups, and there was no significant difference in Aβo levels between younger and older healthy controls. A relatively high amount of Aβo was detected in human brain tissue from AD patients confirming that it could also detect naturally occurring Aβo. Aβo was also detected in the brains from transgenic Tg2576 mice, overexpressing human Aβ, where the Aβo levels decreased with age, which is the opposite to findings on the same mice with a different type of Aβo assay [42]. This might reflect that the two assays detect different populations of Aβo, where the assay used in this paper seems to detect oligomers that are present at highest concentration early in the disease. A potential risk with these kinds of assays is the presence of heterophilic antibodies which could cause a false positive signal, although mainly affecting plasma samples [45]. To show that the Aβo signals was not caused by heterophilic antibodies, a set of CSF samples were spiked with a high concentration of irrelevant mouse IgG, to quench potential heterophilic antibodies, and then measured with the Aβo ELISA. This did not show a decrease in the Aβo levels indicating that the signals were not caused by heterophilic antibodies.

Patients with AD would be expected to have increased concentrations of Aβo since this would reflect the AD pathogenesis with aggregation of Aβ in the brain leading to amyloid plaques. Although it has been reported that the amyloid plaque burden in the brain weakly correlates with the severity of dementia in AD patients [46]. We found higher levels of CSF Aβo in AD patients, although we could not find any correlation between Aβ_1–42_ and Aβo in any of the studies. This would indicate that the lowering of CSF Aβ_1–42_ is not, at least solely, explained by its incorporation into oligomeric forms. The same has been suggested for plasma Aβ and Aβo [44]. Only a small fraction of Aβ_1–40_ and Aβ_1–42_, which are in the high pg/mL to low ng/mL range, seems to be in oligomeric form in CSF, perhaps because the oligomers are stuck in the brain.

We detected that patients with MCI who later converted to AD had increased levels of Aβo compared to controls, while this increase in Aβo was not seen in patients with stable MCI. Although this difference was seen on a group level, many of the samples had lower Aβo levels than could be measured using our Aβo ELISA. The overlap of the Aβo values between the MCI-AD group and control group was substantial and should thus be interpreted with caution. When comparing AD patients that were divided according to their MMSE scores to study how oligomers varied at different stages of AD, we found that AD patients with mild and moderate AD had significantly higher levels of Aβo than controls, while AD patients with severe AD did not differ significantly from the control group. It almost seems as if Aβo levels increase at the onset of the disease when the clinical symptoms appear, and then rises as the disease progresses to later fall back down as the disease gets severe.

To our knowledge no previous study has measured CSF Aβo on several patient materials spanning different stages of AD. In some of the patient materials many of the samples had undetectable or very low levels of Aβo. We cannot explain why the number of patients who had undetectable levels of Aβo varied among the different studies, although there could be some differences in the materials used for sampling CSF at the different centers. There were also some controls in our study who had relatively high levels of Aβo for unknown reason. The controls have been followed up to ensure that they did not develop AD in the near future, minimizing the risk of them having incipient AD although it cannot be fully excluded given that the disease has an onset many years before clinical symptoms [21], [22]. The differences in the patients materials are not likely due to freeze/thawing since we could not detect any loss or gain in Aβo levels when CSF samples were freeze/thawed in five cycles. It has been shown that Aβ is not affected by long term storage [47], although it cannot be completely ruled out that storage conditions might affect the levels of oligomeric forms of the protein. The samples for each study were sampled at one clinical centre and stored in the same way, minimizing possible artifacts from long term storage or differences in sample handling. Even though various techniques have been used to measure CSF Aβo [26], [27], [28], [29], [30], [31], [32], and what was seen in our study, the results seem to remain the same with an increase of Aβo in AD and MCI-AD patients, although with a marked overlap to the controls, resulting in a too weak separation to be considered as a clinical biomarker at this stage. However, as a marker in clinical studies, Aβo can be monitored within patients to measure if CSF Aβo are reduced as an effect of these treatments. This would indicate that the compounds reach their target and reduce the levels of the neurotoxic Aβo.
